# Current status and trend of mitochondrial research in lung cancer: A bibliometric and visualization analysis

**DOI:** 10.1016/j.heliyon.2024.e34442

**Published:** 2024-07-10

**Authors:** Qing Kong, Qingyong Zhu, Yuxia Yang, Wei Wang, Juan Qian, Yong Chen

**Affiliations:** a Functional Examination Department, Northern Jiangsu People's Hospital, Affiliated to Yangzhou University, Yangzhou, 225001, PR China; b Department of Orthopedics and Sports Medicine, Northern Jiangsu People's Hospital, Affiliated to Yangzhou University, Yangzhou, 225001, PR China; c Clinical Medical College, Weifang Medical University, Weifang, 261053, PR China

**Keywords:** Bibliometric analysis, Lung cancer, Mitochondria, Citespace, VOSviewer

## Abstract

This study summarizes and analyzes the relationship between mitochondria and the pathogenesis of lung cancer. The related articles in the Web of Science core literature database are searched and collected, and the data are processed by R software, Citespace, VOSviewer, and Excel. A total of 4476 related papers were retrieved, 4476 articles from 20162 co-authors of 3968 institutions in 84 countries and published in 951 journals. Through various bibliometric analysis tools, the relationship between mitochondria and the pathogenesis of lung cancer was analyzed, the previous research results were summarized, and the potential research direction was found.

## 1 Introduction

As we all know, lung cancer is the leading cause of cancer-related death worldwide [1]. Lung cancer is divided into two pathological subtypes: non-small cell lung cancer (NSCLC) and small cell lung cancer (SCLC). When patients are determined to have lung cancer, they frequently have advanced the disease and miss the window for aggressive surgery since lung cancer is challenging to diagnose in the early stages. Despite the ongoing development of new medical technology, there are relatively few options for the early detection and successful treatment of lung cancer. These include surgery, radiotherapy, chemotherapy, molecular targeted therapy, etc., most of which are just palliative treatments [2]. In addition, the prognosis of lung cancer is poor, and the survival rate of patients with lung cancer has not been significantly prolonged in recent years [3]. Finding the precise pathophysiology of lung cancer from a variety of perspectives is, therefore, crucial to advance lung cancer detection and treatment.

There is evidence that cancer is a mitochondrial related-disease [[4], [5], [6]]. Mitochondria are not only the energy supply factory of cells but also participate in a variety of biological functions of cells, including biosynthesis, reactive oxygen species control, calcium homeostasis, and apoptosis regulation [7]. Cancer cells have much higher bioenergy requirements than normal cells. Since Otto Warburg reported in the last century that tumor cells satisfy energy supply mainly through aerobic glycolysis rather than oxidative phosphorylation, people have reached a consensus on the metabolism of cancer cells. With the deepening of modern scientific research, a new view of this theory has emerged in recent years. Scholars have found that the culprit of tumor high-speed glucose consumption is not cancer cells but the effect of mitochondria on the biological activity of cancer cells [8,9]. Han found that in tumor cells with high rates of oxidative phosphorylation and fatty acid oxidation, mitochondria formed a dense network around lipid droplets. In contrast, in tumor cells with low rates of oxidative phosphorylation, mitochondria were mainly located in the perinuclear, through heavy Plastic cristae and self-structural adaptation to aerobic glycolysis [10]. This finding suggests that mitochondrial metabolism plays a vital role in cancer cells' energy preference and ability to adapt to the metabolic needs of different subtypes of cancer cells. In addition, cancer cells' mitochondria also exhibit other behavioral changes, such as mitochondrial quality control systems and dynamics [11]. Mitochondria are in a dynamic cycle of fusion and fission for a long time. This dynamic cycle is indispensable for the homeostasis of mitochondria and can maintain the balance of mitochondrial mass in cells. This dynamic cycle of mitochondrial fusion and fission is called mitochondrial dynamics. In addition, mitochondria can selectively engulf damaged mitochondria through a unique mitophagy pathway to maintain normal mitochondrial metabolism in cells [12]. Together, the above mechanisms achieve homeostasis of mitochondrial quality and maintain the normal function of cells. Some studies have found that lung cancer tumor cells have different levels of changes in mitochondrial dynamics and autophagy, and artificial changes in mitochondrial dynamics will directly lead to changes in tumor behavior. Increased mitochondrial fission in cancer cells may lead to rapid tumor cell proliferation and tissue infiltration, while increased fusion may make cells resistant to physical toxicity and stressful environments [13]. Currently, no drugs target mitochondrial metabolism or other mitochondrial properties in lung cancer treatment, but this is a potentially exciting research direction for future interventions in lung cancer.

Since 2010, the number of studies involving mitochondrial behavior changes in lung cancer cells has increased dramatically. However, due to the large number of articles, uneven quality, and diversity of research methods, there is currently a lack of summary of the latest research progress on mitochondrial behavior changes in lung cancer cells and generalizations. Therefore, a scientific research method is needed to evaluate the key research results of mitochondrial research in lung cancer research and to provide research ideas for follow-up research.

The scholar Paul Otlet first put forward the scientific method of bibliometric analysis in 1934, believing that it is a method to help scholars quickly discover hot topics in a particular field and reveal their development trends [14]. Bibliometric analysis can assist researchers in swiftly grasping the state of the research field as a whole and assessing the caliber of published research findings in order to identify the research field's frontier and future development path [15,16]. In bibliometric analysis, the commonly used methods include citation analysis, cluster analysis, correlation analysis, and so on [17]. Bibliometrics has been a popular tool in scientific research in recent years. The mitochondria of lung cancer cells have not yet been the subject of a bibliometric investigation by researchers. As a result, this study aims to visually assess the research on lung cancer cells' mitochondria, characterize the state of this field's research, and highlight the contributions and connections across different nations, institutions, and authors.

Exploring the disease's occurrence and development mechanism is essential in clinical-oriented transformation. In this paper, we searched the Web of Science Core Library (WoSCC) for articles on lung cancer and mitochondrial research in the past 20 years. To comprehend the most recent research hotspots and development trends of lung cancer mitochondrial research and to look ahead to the future research direction, the retrieved findings were screened, summarized, graphically evaluated, and exhibited in the form of charts and charts.

## 2 Methods

### 2.1 Data sources and search strategies

WOS is the most commonly used literature retrieval database for scholars, including a wide range of high-quality journals and comprehensive citation records [15,18]. In order to ensure that the retrieved data is comprehensive, this study retrieves and downloads the data through WoSCC's Science Citation Index Expanded (SCI-EXPANDED) and SSCI. We have systematically searched the articles whose types are articles and review up to April 1, 2023. The language is set to English, with no nationality and major restrictions. The retrieval strategy is as follows: TS=("lung neoplasm" OR "pulmonary neoplasm" OR "lung cancer" OR "lung tumor" OR "cancer of lung" OR "lung adenocarcinoma" OR "lung squamous cell carcinoma" OR "adenocarcinoma of the lung" OR "bronchial neoplasm" OR "Pancoast syndrome" OR "non-small cell lung cancer" OR "NSCLC" OR "non-small cell lung carcinoma" OR "non-small lung cancer" OR "none small cell lung cancer" OR "large cell lung cancer" OR "SCLC" OR "small-cell lung cancer" OR "small-cell lung carcinoma") AND TS=(mitochondrion OR mitochondria OR mitochondrial). We export and save all search results from WOS in plain text format.

### 2.2 Data collection

On April 1, 2023, we downloaded and acquired raw data from WoSCC to prevent data bias. The "bibliometrixpackage4.0.1″ of R software (version 4.2.1) is a bibliometric analysis and data collection tool. We adhere to the instructions and import the data into R software for primary storage and cleaning [19]. We introduce essential data into Microsoft Office Excel, such as the title, author, year, country, institution, journal, keyword, and so forth (version 2019).Due to the large workload, three authors were involved in this data processing at the same time.

### 2.3 Bibliometric analysis

We can more effectively extract the findings of bibliometric analysis by scientific drawing and visual analysis, and this procedure calls for the involvement of various tools. We compute the percentage of papers published annually, the total number of citations, and the average number of sources using Excel through the R software statistics of the author, organization, country, and other information. VOSviewer (version 1.6.18) and Citespace (version 6.2.1. R3) are two different kinds of software for data analysis and visualization. These two software are used for coupling network construction and visualization analysis among journals, countries, co-authors, and keywords, identifying citation outbreak keywords, constructing density visualization maps of co-citation references, and drawing journal double map superposition and national cooperation network maps.

## 3 Results

### 3.1 Overview of publications

**Fig. 1:**
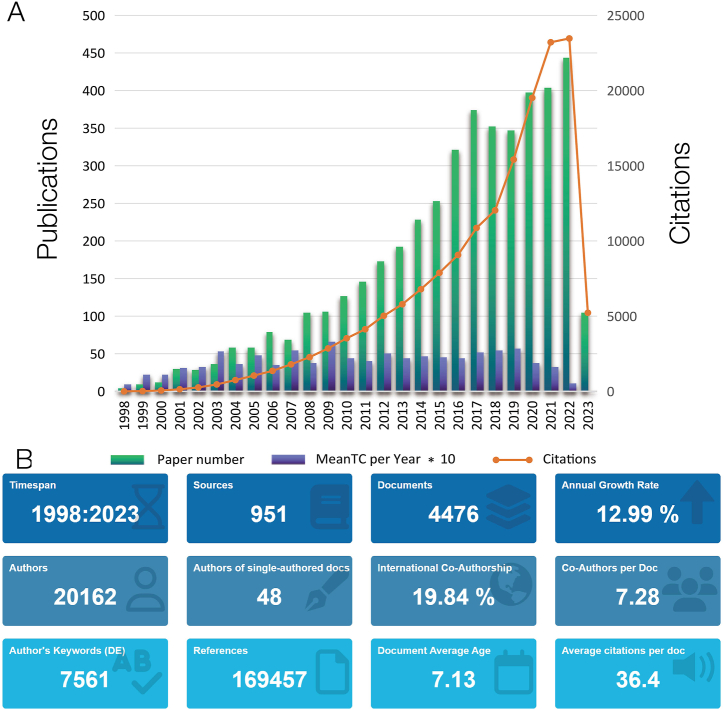
Analysis of published papers. (A) Year distribution and citations of mitochondrial research in lung cancer. (B) General information about papers related to mitochondrial research in lung cancer.

We retrieved 4476 articles using the method described in the method section (Fig. 1A). The period of these articles is from 1998 to 2023, and the number of published articles increases yearly. The year is concentrated between 2016 and 2022, indicating that the research on mitochondria of lung cancer cells has become a research hotspot in recent years. The year with the highest number of citations is 2022. 20162 co-authors from 3968 institutions in 84 countries contributed to the 4476 pieces, which were then published in 951 publications. These publications referenced 169457 other articles from 10141 journals (Fig. 1B). The top 10 papers cited are shown in Table 1, among which the three most frequently cited articles are "Targeting cancer cells by ROS-mediated mechanisms: a radical therapeutic approach?" published by Trachootham, D in the journal Nat Rev Drug Discov in 2009, and have been cited 3748 times so far. The second is Galluzzi, L. in 2012, "Molecular Mechanisms of cisplatin resistance," published in Oncogene, and the third is Fulda, S in 2010, " Targeting mitochondria for cancer therapy published " in Nat Rev Drug Discov.

**Table 1:** The top 10 most cited papers related to mitochondrial research in lung cancer.

Ranking	Title	Journal	First author	Publishing year	Citations	IF (2022)
1	Targeting cancer cells by ROS-mediated mechanisms: a radical therapeutic approach?	Nat Rev Drug Discov	Trachootham, D	2009	3748	112.288
2	Molecular mechanisms of cisplatin resistance	Oncogene	Galluzzi, L	2012	1746	8.756
3	Targeting mitochondria for cancer therapy	Nat Rev Drug Discov	Fulda, S	2010	1230	112.288
4	A mitochondria-K+ channel axis is suppressed in cancer and its normalization promotes apoptosis and inhibits cancer growth	Cancer Cell	Bonnet, S	2007	1189	38.585
5	Mitochondrial metabolism and ROS generation are essential for Kras-mediated tumorigenicity	Proc Natl Acad Sci U S A	Weinberg, F	2010	1150	12.779
6	Understanding the Intersections between Metabolism and Cancer Biology	Cell	Vander Heiden, MG	2017	1136	66.85
7	The CoQ oxidoreductase FSP1 acts parallel to GPX4 to inhibit ferroptosis	Nature	Bersuker, K	2019	994	69.504
8	From selenium to selenoproteins: Synthesis, identity, and their role in human health	Antioxid Redox Signal	Papp, LV	2007	954	7.468
9	Targeting mitochondria metabolism for cancer therapy	Nat Chem Biol	Weinberg, SE	2015	916	16.174
10	One-Carbon Metabolism in Health and Disease	Cell Metab	Ducker, GS	2017	863	31.373

### 3.2 Visualization analysis of keyword co-occurrence

**Fig. 2:**
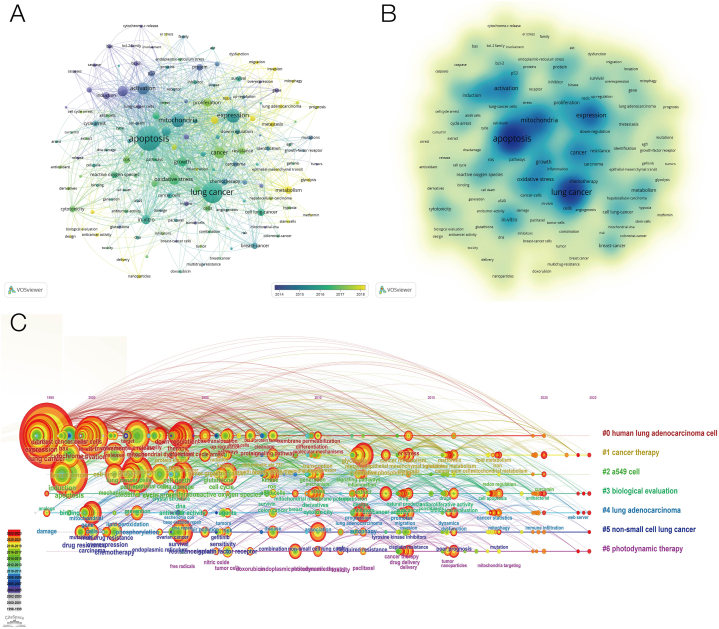
Keywords related to mitochondrial research in lung cancer. (A) Visualization of keywords drawn by the VOSviewer. The color of the circles indicates the average year of publication. (B) Density visualization map of the keywords. The size and color depth of the circle indicates the frequency of occurrence. (C) Timeline graph of keywords related to lung cancer and epigenetic research between 1998 and 2023. Circle size represents the frequency of occurrence, with red representing more recent occurrences. (For interpretation of the references to color in this figure legend, the reader is referred to the Web version of this article.)

Keywords can represent the theme and main points of the paper and, through the reproduction of keywords, can describe the research hotspots and trends in the field. We used VOSviewer to visually analyze the keywords that appeared 50 times or more in all articles and got a total of 143 keywords. According to Fig. 2A and B, the larger the circle represented by the keyword and the darker the keyword color, the higher the frequency of occurrence and the more representative the research hotspot in the field; different colors represent the average time and year in which the keyword appears. It can be observed that the five most frequently used keywords in the field of lung cancer mitochondrial research are "apoptosis," "lung cancer," "expression," "mitochondria," and "activation." With the increase of time order, the color changed from blue to yellow, and the keywords "metastasis," "metabolism," "mitophagy," and "dysfunction" appeared later, indicating that a new direction has emerged in the field of mitochondria. Keywords change over time, as shown in Fig. 2C; the node size represents the frequency of recent occurrences, and the color represents the average time of the event. A total of six clusters were separated, including "human lung adenocarcinoma cell," "cancer therapy," "a549 cell", "biological evaluation," "lung adenocarcinoma," "non-small cell lung cancer," and "photodynamic therapy".

**Fig. 3:**
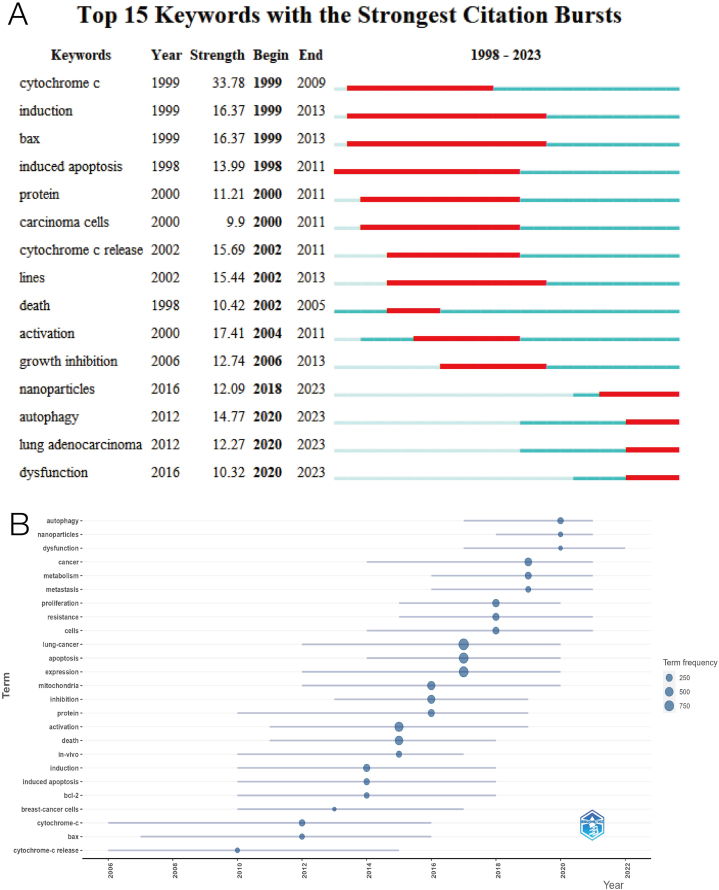
(A) Top 15 keywords with the most substantial citation bursts of mitochondrial research in lung cancer. (B)Trends in research topics over time.

Explosive words can show the research hotspots and trends that appear in a certain period. We first calculate the intensity of the top 15 explosive words from 1998 to 2023 through Citespace software (Fig. 3A). The intensity of these 15 explosive words is almost above 10, and the most intense keyword is "cytochrome *c*". In recent years, the explosive words are "autophagy", "lung adenocarcinoma" and "dysfunction". In addition, we calculate the Trends topics by R software. Fig. 3 displays the top 25 Trends topics with blue lines denoting occurrences duration and circle size denoting usage frequency. Similar to outbreak words, keywords related to mitochondrial dysfunction and mitochondrial autophagy have increased in recent years (Fig. 3B).

### 3.3 Analysis of journals

**Fig. 4:**
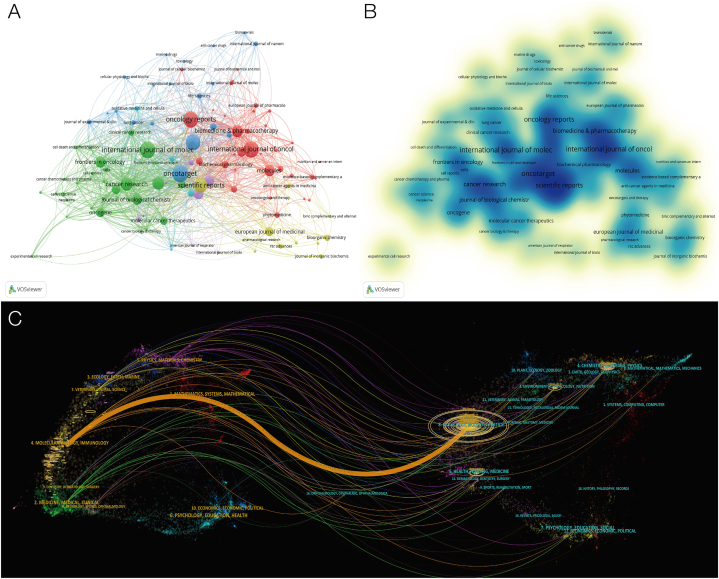
Analysis of journals. (A) Mapping of cooperative networks between journals. (B) Density visualization map of the journals. (C) Double image overlay of journals.

The top 10 cited journals for the top 100 publications on the subject of lung cancer and mitochondria research are displayed in Table 2. "NATURE REVIEWS DRUG DISCOVERY" is the most cited, followed by "CANCER RESEARCH", and the journal with the highest average citation is "NATURE REVIEWS DRUG DISCOVERY", indicating that this journal has great influence in the research of lung cancer mitochondria. Citation analysis allows us to see which publications and articles are often referenced in this area of study. We use VOSviewer to create a citation map for publication and set a minimum citation criterion of 10. The 105 journals considered for the examination of citations are depicted in Fig. 4A and B. The circle size and blue depth represent the number of journals published. Additionally, we examine the relationship between the citation journal and the cited journal using the double overlay map of the journal (Fig. 4C). The citing journal is represented on the left, the cited journal is represented on the right, and the colored path shows the link between the two citations. The image primarily demonstrates that publications in molecular science, biology, and immunology frequently mention articles in those same three fields of study, as well as in genetics.

**Table 2:** The top 10 journals with the most citations of mitochondrial research in lung cancer.

Ranking	Journals	Citations	Documents	Average Citation/Publication
1	NATURE REVIEWS DRUG DISCOVERY	4978	2	2489
2	CANCER RESEARCH	4853	61	79.6
3	ONCOGENE	4339	44	98.6
4	CANCER CELL	3911	11	355.5
5	CELL METABOLISM	3687	10	368.7
6	JOURNAL OF BIOLOGICAL CHEMISTRY	3627	48	75.6
7	CLINICAL CANCER RESEARCH	3272	29	112.8
8	CELL DEATH&DISEASE	3170	62	51.1
9	ONCOTARGET	2832	83	34.1
10	PLOS ONE	2550	77	33.1

### 3.4 Network visualization map of co-citation references

**Fig. 5:**
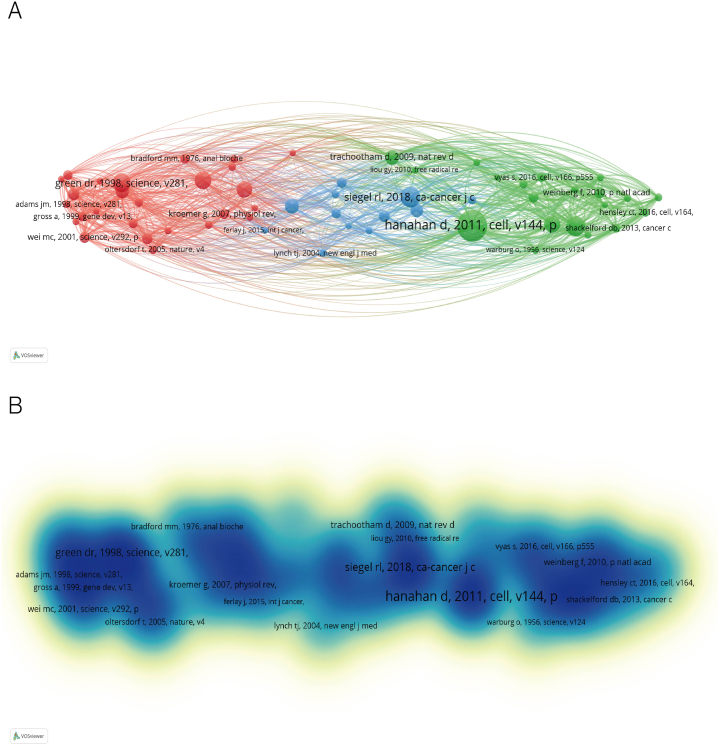
Visualization mapping of co-citation references. (A) Network visualization map. (B) Density visualization map.

Two published articles will be associated if they both appear in the reference list of a different published article at the same time. The greater the number of co-citations, the greater the influence of the publication. The co-citation map of the literature is also created using VOSviewer, with a minimum threshold of 50 citations. A total of 58 kinds of literature are utilized for the co-citation study. The reference network in which the publication is concurrently mentioned is depicted in Fig. 5A. A document is represented by each circle. The more frequently the literature is quoted, the more comprehensive the circle. Fig. 5B's darker shades more clearly display the literature with more co-citations.

### 3.5 Analysis of countries

**Fig. 6:**
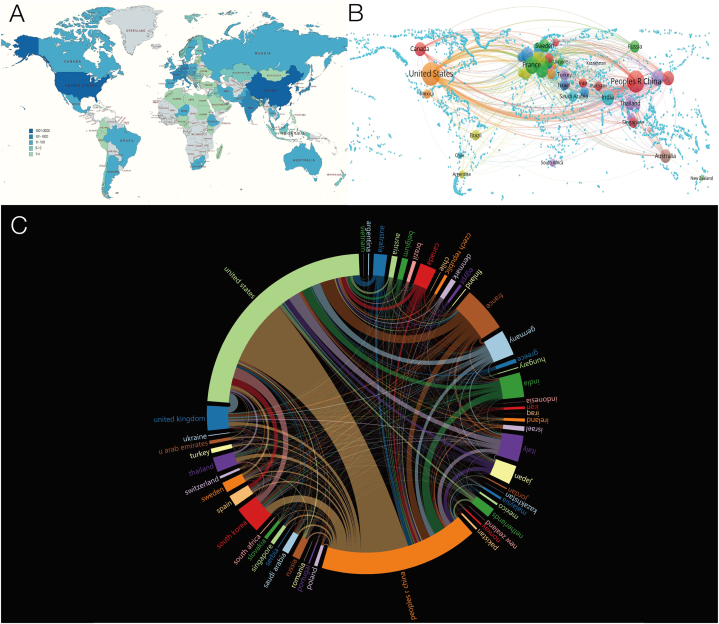
Distribution and linkage of country composition related to mitochondrial research in lung cancer. (A) Distribution of publications by country. (B) Visualizing networks for international research collaboration. (C) Chord diagram for assessing international cooperation networks.

Among the 4476 publications in the field of lung cancer mitochondrial research, a total of 80 countries are distributed, as shown in Fig. 6A. The number of statistical countries is not limited to the first author and correspondent authors, of which China has the highest number of articles, reaching 2036, followed by 1001 in the United States and 336 in South Korea. The countries with the highest number of citations are the United States, China, and France, which have been cited 66642 times, 46826 times, and 13296 times, respectively. Fig. 6B and C shows the visualization network of international cooperation, and the circle and chord area size represent the number of citations. International cooperation can quickly promote the progress of research. It can be observed that most of the cooperative relations are related to the United States, and the cooperation with China is relatively close, while the cooperative relationship between other countries is relatively weak, indicating that international cooperation in lung cancer mitochondrial research mainly revolves around the United States, China, France, and other countries.

### 3.6 Analysis of organizations

**Fig. 7:**
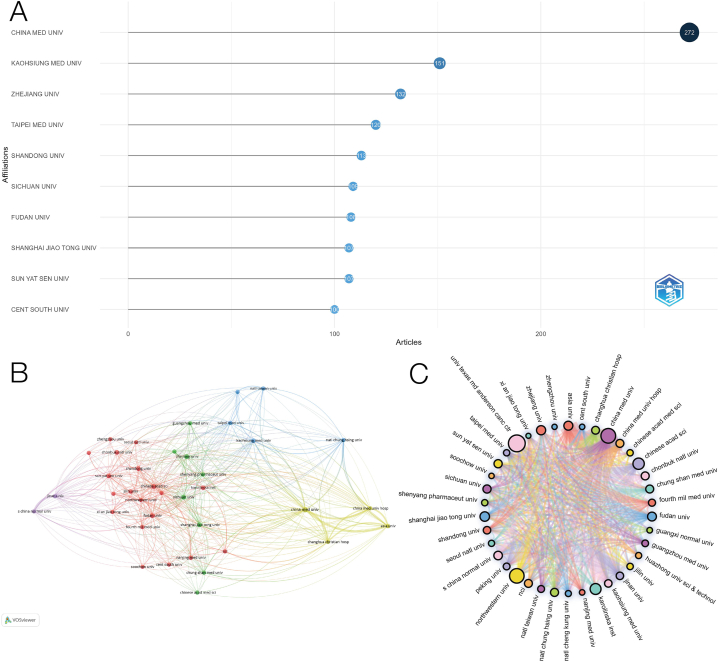
Analysis of co-authorship organizations. (A)The 10 most relevant institutions among publications. (B) Co-authorship of the institutional network visualization map. (C) Mapping of cooperative networks.

The ten most relevant organizations are shown in Fig. 7A. According to statistics, a total of 3968 organizations were engaged in the contribution. Among them, CHINA MED UNIV contributed the most, with 272 articles, followed by KAOHSIUNG MED UNIV and ZHEJIANG UNIV, with 151,132 articles respectively. The majority of important institutions belong in the United States. Fig. 7B and C shows the partnership between organizations, and the circle size represents the number of times cited. We created a cooperative relationship between 38 entities and set the minimum collaboration at 30, which has more cooperation with CHINA MED UNIV, CHIN ESEA CAD SCI, and ZHEJIANG UNIV. It can be observed that most of the cooperation between organizations is carried out in our country, among which CHINA MED UNIV CHI, N ESEA CAD SCI, and ZHEJIANG UNIV are the most cooperative institutions in China. In addition, there are some American institutions, such as NORTHWESTERN UNIV and UNIV TEXAS MD ANDERSON CANC CTR, which have a high number of citations and have a certain degree of cooperation with institutions of various countries.

### 3.7 Analysis of authors

**Fig. 8:**
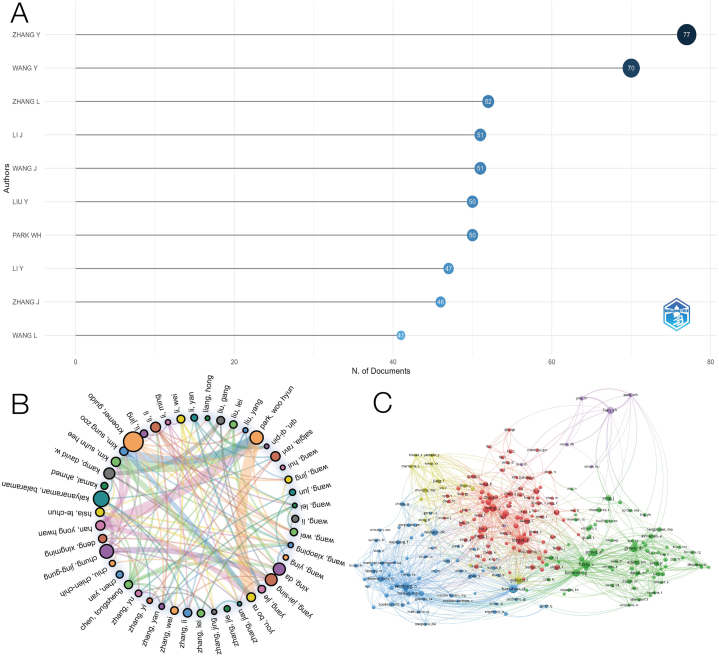
Analysis of co-authorship organizations. (A)The ten most relevant institutions among publications. (B) Co-authorship of the institutional network visualization map. (C) Mapping of cooperative networks.

A total of 24705 writers contributed to the work through data collection, and the top 10 most pertinent authors, as shown in Fig. 8A, are mostly researchers in the field of lung cancer. Among them, ZHANG, Y is the most relevant author among the top 100 articles, with a total of 77 articles, followed by WANG, Y, with 70 articles. With a minimum of 10 author collaborations, Fig. 8B shows the co-author network of several closely related authors, indicating that the collaboration between authors is carried out around Kroemer, G, Kalyanaraman, B, and Park, WH. Fig. 8C shows the network of co-cited authors: the minimum number of contacts between authors is 50. A total of 254 authors are included in the network, and five different author clusters are separated. The circle size represents the number of times it is cited.

### 3.8 Analysis of citation

Among the top ten papers cited most frequently in this search, the most frequently cited article was a review of ROS-mediated targeted therapy in 2009. Through the summary of previous studies, it is concluded that cancer cells can be eliminated by regulating the unique redox regulation mechanism of cancer cells. In 2010 and 2016, there was a review of the current progress of mitochondria in cancer treatment. The remaining articles discussed the drug resistance mechanism of cisplatin-based on mitochondrial apoptosis, the anticancer mechanism of dichloroacetic acid by inhibiting mitochondrial pyruvate dehydrogenase, the interruption of mitochondrial function caused by the deletion of mitochondrial transcription factor A gene, apoptosis-inducing factor Mitochondrial 2 is a kind of iron death resistance factor, and the basic principle of mammalian carbon metabolism was reviewed. It is a new research direction to discover the biological importance of mitochondrial carbon reactions.

## 4 Discussion

Cancer has always been a major health problem for human beings. Worldwide, the morbidity and mortality of lung cancer remain high, bringing a huge economic burden and threatening the health of human beings all over the world [20]. Although there are a variety of treatments for lung cancer, most of them are palliative [21,22]. Exploring the mechanism of the occurrence and development of lung cancer is helpful in furthering the characteristics of lung cancer cells, finding new therapeutic targets, and providing a new target for the precise treatment of lung cancer.

The incidence of lung cancer remains high, which has the highest incidence of cancer in the world. The symptoms of lung cancer are not typical; most of the patients are in the late stage of the disease when they are found, the prognosis is poor, and the survival time is short. Lung cancer is a complex disease involving multiple factors such as genetics and environment, and a variety of theories have been formed in the course of continuous research by scholars for many centuries [23]. The relationship between mitochondria and lung cancer mechanism was first discovered by OttoWarburg in the last century, and for more than a hundred years, new mitochondrial research targets have been developed. In recent years, researchers have published a sharp increase in results. In order to clarify the development trend in this field, it is very important to summarize and analyze articles in this field accurately.

Bibliometric analysis is a scientific method that can accurately and simply analyze the research hotspots and development status of a certain scientific field. It originated in the field of informatics, and the development of the big data era of follow-up has been gradually applied to various fields. At present, it has become an independent science major and become an important research means in different scientific fields [19]. In this study, we used its commonly used software to comprehensively evaluate the authors, institutions, countries, keywords, citations, and their links to articles on the relationship between mitochondria and lung cancer mechanisms. The data are clustered and iterated by mathematical and statistical methods, and the number, citation times, and correlation of academic achievements are quantitatively analyzed.

Among all the related literature, the time span is from 1998 to 2013, and the amount of literature has increased rapidly since 2016, indicating that mitochondria have been paying more and more attention to mitochondria in the study of lung cancer in recent years. The literature with high citation frequency shows that the achievement has extensive influence and high recognition in the field. In this metrological analysis, there are 6 articles cited more than 1000 times, and the top 10 articles cited are mostly distributed in top journals such as "Nat Rev Drug Discov", "Cancer Cell", "Cell" and "Nature". The article "Targeting cancer cells by ROS-mediated mechanisms: a radical therapeutic approach?" written by Trachootham, D, in 2009, has the highest number of citations. By April 1, 2023, the number of citations has reached 3748. In this paper, the authors summarized the genetic and reactive oxygen species changes in cancer cells, and mitochondria are one of the important sources of reactive oxygen species. Cancer cells may enhance their drug resistance by adapting to oxidative stress [24]. Other highly cited articles also discuss some very important points. The author Galluzzi, L discussed in Molecular Mechanisms of Cisplatin Resistance that the most prominent mechanism of action of platinum anticancer drugs (best known as cisplatin) is to kill tumors through DNA damage and mitochondrial apoptosis, but cancer cells may also increase mitochondrial resistance to chemical drugs [25]. Interestingly, the first two articles have discussed that cancer cells may carry out drug resistance through mitochondria. In contrast, the author Fulda, S discussed in Targeting Mitochondria for Cancer Therapy that because cancer cells have mitochondrial structure and metabolism different from normal cells, some lethal signal transduction pathways are also involved, which can induce apoptosis through the mitochondrial membrane or mitochondrial metabolism. Mitochondria and key organelles targeting the death of cancer cells were proposed. Among the articles cited in the top 10, more articles describe the relationship between mitochondrial reactive oxygen species and oxidative stress and lung cancer cells. Most of these articles were published before 2010.

As a carrier of research results, journals can help disseminate important discoveries in this field, and high-quality journals can often bring more meaningful results. Most of the articles included in the study were published in cancer-related journals, among which the most cited journal was "Nat Rev Drug Discov", which only published two articles, and the average citation of a single article was as high as 2489 times, much higher than other journals. Although some journals published a high number of articles, "Oncotarget" and "Plosone" participated in the publication of 83 and 77 articles, the average single article citations were low, only 34 and 33 times, and some journals such as "Cancer cell" and "Cell mettab" published articles Although there are only 11 and 10 articles respectively, the citations of a single article reached 355.5 and 368.7 times, indicating that these journals have an important position in this research field.

It can be clearly seen from Fig. 6 that China and the United States are the main contributors to these published documents, followed by countries such as South Korea, India, and Japan. China published a total of 2036 articles with a total of 46826 citations, and the United States published 1001 publications with a total of 66642 citations. In addition, although France published 124 papers, it was cited 13,296 times. In addition, it can be seen from the visual analysis that the United States is the center of international cooperation and has many connections with other countries. The results suggest that the cooperation among developed countries is very close, such as the cooperation between China, France, and South Korea, while other countries have less contact in this regard, which suggests that for the sake of rapid progress in related fields, it is very important to further strengthen international cooperation. Since China is the country with the largest number of publications on lung cancer mitochondrial research, most of the most relevant institutions are from China, and the most relevant institution is CHINA MED UNIV, which participated in the publication of 272 papers. The most frequently cited organization is UNIV PARIS 5 from France. Most of the inter-agency cooperation occurs in Chinese institutions, and some excellent publishers with more cited times have relatively weak cooperative relations with other institutions. In the analysis of the information of the authors who have published papers in related fields, we find that there is close cooperation between those excellent authors, while there is relatively little cooperation between other authors. The above data show that the international and institutional distribution of research in this field is uneven. The above data show that the international and institutional distribution of research in this field is uneven.

Many scholars have made outstanding achievements in this field in the study of mitochondria of lung cancer, such as Guido, K, a scholar from the University of Paris. The research results of Guido, K are included in 1668 articles in the WOS database, which has maintained a stable output of scientific research achievements for many years, with a total frequency of 242064 citations and an h-index of 231. Lorenzo, G from Yale University, is also a very influential writer. WOS collected a total of 382 articles contributed by him, with a total of 64129 citations, with an h index of 111. The authors are widely cited in cross-disciplinary oncology and cell biology and are known as "highly cited researchers in molecular biology and genetics". In addition, many outstanding authors work in lung cancer, biology, or genetics. There is a close cooperative research model among these excellent authors. At the same time, it also shows that the research in this field is unevenly distributed worldwide.

Through the analysis of keywords, scholars can find hot spots in the research field and explore new research directions. The most frequently used keywords that can be observed in Fig. 2 include lung cancer, apoptosis, mitochondria, and expression. Some keywords that have been used frequently for a long time are bcl-2, caspase, cytochrome-c, and so on. These keywords are related to apoptosis. In recent years, some new keywords have emerged, such as metabolism, mitophagy, dysfunction, and nanoparticles, indicating that scholars pay more attention to metabolism, kinetics, or targeted therapy. Among the topic trends we counted, we found that the longer timeline involved in the mechanism was cytochrome-c, and the number of results in this direction decreased sharply after 2016. Cytochrome-c is known to exist in mitochondria and is a key participant in the process of ATP synthesis. When cells receive specific stimuli, cytochrome-c is released into the cytoplasm, which leads to programmed cell death by inducing apoptosis [26,27], indicating that apoptosis-related research has been the focus of mitochondrial research in lung cancer for many years. In the articles we searched and included in the study, scholars studied the apoptosis pathway of cancer cells in different aspects. Scholars mentioned the correlation between ROS-related oxidative stress and cancer cell apoptosis [[28], [29], [30]], or the relationship between mitochondrial bioenergy disorders and cancer cell apoptosis [7,8,31,32]. They discussed the target of cancer therapy [33], and cancer cell survival and drug resistance through metabolic or apoptotic pathways during cancer treatment [34,35]. In recent years, the research on the related targets of mitochondrial dynamics has increased. Scholars have studied lung cancer through mitochondrial fusion, division, and autophagy, which provides a theoretical basis for the future development of anticancer drugs based on mitochondrial dynamics [[36], [37], [38], [39]]. At the same time, with the advancement of science and technology, the goal of more research is to optimize drug targets, improve drug pharmacology, and achieve precise treatment of lung cancer [40]. The combination of research and new technologies, such as Zhang L and other scholars, analyzed the differences in gene amplification, cell composition, and expression modules between the two main subtypes of lung adenocarcinoma and squamous cell carcinoma [41], through Integrated single-cell RNA sequencing technology, through multi-omics (Genome, Epigenome, Transcriptome, and Proteome) for comprehensive analysis of lung tumor prediction and treatment [42,43], development of nanomedicine targeting mitochondrial respiration or function to inhibit the progression of lung cancer [[44], [45], [46]].

The study of mitochondrial metabolism in lung cancer runs through the study, and the detection of mitochondrial metabolism is involved in a large number of studies [47,48]. Otto Warburg initially proposed that mitochondrial respiratory defect is the fundamental basis of aerobic glycolysis and carcinogenesis. After decades of scientific development, it has been found that not all tumors have the characteristics of aerobic glycolysis, and mitochondrial respiratory defect is not the root cause of aerobic glycolysis and tumorigenesis [49]. Some conventional ideas are being gradually updated by studies published in recent years. Although cancer cells mainly rely on glycolysis, many cells have fully functional mitochondria, indicating that mitochondria are fragile target organelles in cancer cells. Therefore, a key difference between cancer and normal cell metabolism is metabolic reprogramming [50]. A recent high-quality study used a comprehensive platform of positron emission tomography, respiratory measurement, and three-dimensional scanning block electron microscopy to analyze the structure and function of mitochondrial networks and bioenergy phenotypes in non-small cell lung cancer (NSCLC). Multiple bioenergy phenotypes, metabolic dependence, and the existence of mitochondrial networks divide NSCLC into different subgroups [51]. The spatial metabonomics technology developed in recent years is also helpful in detecting the metabolic spatiotemporal information in the anticancer process of drugs, determining the toxic dose exceeding the therapeutic effect, and exploring the mechanism of related cancer pathology [52]. Understanding different functional levels of mitochondria in the future will not only provide new insights into the discovery of different subtypes of tumors but also provide a way for targeted mitochondrial metabolism in the treatment of lung cancer. Some evidence suggests that mitochondrial small molecular inhibitors targeting OXPHOS inhibit tumor proliferation and growth [53]. Some tumor cells sensitive to low glucose and low mitochondrial oxidation ability are sensitive to biguanide drugs that effectively inhibit mitochondrial complexes [54].

Reactive oxygen species (ROS) is a natural metabolic by-product of aerobic mitochondrial metabolism under normal physiological conditions [55]. Excessive ROS can cause oxidative damage to cell proteins, lipids, and nucleic acids, leading to cell death. ROS also participates in the execution of programmed cell death through peroxidation of cardiolipin, which releases cytochrome *c* from the inner membrane of mitochondria [56]. Glutamine is the most abundant amino acid in plasma and the main source of reducing nitrogen in cells. Glutamine is produced by a metabolic pathway of glutamine mitochondria. Some studies have found that cancer cells show abnormally high glutamine consumption and glutamine decomposition rate [57]. Some studies have found that the combination of glutaminase inhibitors and radiotherapy in NSCLC cell lines can improve the response to radiotherapy [57]. Mitochondrial-targeted antioxidants have been found to be effective in cancer prevention and anticancer therapy [58].

Studies on mitochondrial dynamics have increased significantly since 2013. the processes of mitochondrial fusion and division control cell cycle, metabolism, and survival, which are related to a wide range of physiological and pathological conditions [59]. There is a lot of evidence that lung cancer cells change mitochondrial dynamics to gain proliferation and survival advantages [[60], [61], [62]]. The keyword mitochondrial autophagy began to appear in 2017, and 2017 has been a hot period for mitochondrial autophagy in lung cancer. There is a lot of evidence that mitochondrial autophagy is a key factor leading to drug resistance in cancer cells. There are some excellent research results; m6a methyltransferase METTL3 leads to drug resistance in small-cell lung cancer by targeting the mitochondrial autophagy pathway [63]. PINK1-mediated mitochondrial autophagy can promote oxidative phosphorylation and redox homeostasis and induce drug resistance persistence in cancer cells [64].

Mitochondrial dynamics disorders are associated with advanced lung cancer and shorter survival in the late stage. The content of mitochondrial DNA and the expression level of key proteins can provide important information for clinical diagnosis. Damage to mitochondrial DNA leads to mitochondrial dysfunction, which is a key mechanism in lung cancer. In 2017, Fangming Liu [65] Pointed out in the article that mitochondrial DNA mutations may participate in the pathogenesis of lung cancer by altering proteins, leading to impaired respiratory chain function, increasing mitochondrial oxidative stress, generating free radicals, reducing cellular energy capacity, and regulating electrochemical gradient-controlled cell apoptosis. Meanwhile, changes in mitochondrial DNA content were also found in lung cancer, Hui Xu [66]found that the copy number of mitochondrial DNA in lung cancer tissue decreased and was associated with a shorter survival time in the late stage of the disease. Mitochondrial DNA variation has been found more and more and is expected to become a potential biomarker for monitoring the severity, duration, stage, treatment response, and prognosis of patients with lung cancer. Mitochondrial dynamic protein-associated protein (Drp-1) is a key factor in regulating mitochondrial fission. The study shows that the level of Drp-1 in NSCLC decreases [67], The low expression of Drp-1 is associated with advanced lung cancer, which indicates that the low level of Drp-1 is also related to the progression of lung cancer. Drp-1 increases genomic instability, radiation, and drug resistance in lung adenocarcinoma [68]. Mitochondrial Drp-1 is related to the proliferation, invasion, and metastasis of lung adenocarcinoma cells. Inhibition of Drp1 expression may be helpful to the anti-tumor therapy of lung cancer [69].

In the future, a variety of important roles of mitochondria will also be used to design new mitochondrial-targeted anticancer drugs. Cancer stem cells have unique characteristics, which make them vulnerable to mitochondrial-targeted drugs [70]. Chemotherapy is very important in the systemic treatment of lung cancer. However, chemotherapy can also lead to drug resistance, low selectivity, and low side effects on cancer cells. Therefore, targeted drug delivery or treatment to mitochondria contributes to the specificity and toxicity of traditional anticancer methods. At present, some mitochondrial targeting nano-carriers based on lipophilic cations have been developed [71], mitochondrial targeting Nano-carriers based on Peptide [72], mitochondrial targeting nano-carriers in photodynamic therapy [73]. In the past few years, it has shown great potential for cancer treatment, and some studies have carried out preclinical and clinical trials [74,75]. Targeted therapy of tumor microenvironment (TME) is becoming a key research field. Tumor microenvironment refers to non-cancer cells and components in tumors. Intercellular communication between TME and cells promotes the progression of cancer and affects the response to existing treatments [76]. Cells in TME undergo various biological processes of metabolic reprogramming, oxidative metabolism, and glycolysis, and mitochondria play an important role [77]. It is an effective therapeutic strategy to link the changes in mitochondrial metabolism with the activation of immune cells in TME. The accumulation of mitochondrial targeting drugs based on triphenylphosphine in tumor cells is more effective than in normal cells. It not only inhibits cancer cell proliferation by affecting mitochondrial function but also affects the anti-tumor immunity of immune cells in TME[78]. A new type of zero-valent iron nanoparticles showed the effect of targeting TME and cancer cells. Iron death of cancer cells was induced by mitochondrial dysfunction, and anti-tumor immunity was enhanced by transferring pre-tumor M2 macrophages to anti-tumor M1, resulting in significant inhibition of tumor growth and metastasis, showing sufficient therapeutic potential [79].

Because the article we analyzed is only from the WOS, there may be some excellent research results not included in it, and some of the most cutting-edge research results may not be published, which will affect the summary and prospect of this article on the relationship between mitochondria and lung cancer mechanism. The solution to these problems is to pay continuous attention to the relevant articles and analyze and deal with them in a timely manner. In addition, it is undeniable that there may be repeated counting of nodes in the data analysis through the software, and there may also be some different calculation parameters between each software, and some of the data cannot accurately represent the influence of the paper. As lung cancer is also divided into different subtypes, which represent different heterogeneity, the mitochondrial differences of different lung cancer heterogeneity need to be further explored in the future.

## 5 Conclusion

Generally speaking, this paper is the first comprehensive bibliometric and visual analysis of the achievements in the field of lung cancer mitochondrial research through a variety of bibliometric analysis tools, including Citespace, VOSviewer, R software, and Excel. Through constantly adjusting the retrieval strategy and searching many times, more than 4476 articles collected so far basically cover the research related to mitochondria in lung cancer diseases. In the past ten years, mitochondria have gradually become the focus of scholars, and the number of studies has increased sharply. In addition, more studies in the future will focus on most cancers that do not conform to Warburg's early hypothesis that mitochondrial functional defects are carcinogenic. In these tumors, targeting mitochondria for early diagnosis or treatment of cancer may bring new hope to patients.

## Data availability statement

The data covered in this article is available from the corresponding author.

## CRediT authorship contribution statement

**Qing Kong:** Writing – original draft, Conceptualization. **Qingyong Zhu:** Writing – review & editing, Writing – original draft, Conceptualization. **Yuxia Yang:** Formal analysis, Data curation. **Wei Wang:** Visualization, Data curation. **Juan Qian:** Validation, Software. **Yong Chen:** Conceptualization.

## Declaration of competing interest

The authors declare that they have no known competing financial interests or personal relationships that could have appeared to influence the work reported in this paper.
